# The magnet species effect of two-leaf squill (*Scilla* spp.) on pollinator competition with the snowdrop (*Galanthus nivalis* L.)

**DOI:** 10.3389/fpls.2026.1760796

**Published:** 2026-01-30

**Authors:** Pavol Prokop, Fedor Čiampor, Olena Bielikova, Ladislav Pekárik, Tomáš Čejka, Zuzana Provazník, Michaela Mešková, Viktória Vanerková, Božena Šerá, Zuzana Čiamporová-Zat'ovičová

**Affiliations:** 1 Department of Environmental Ecology and Landscape Management, Faculty of Natural Sciences, Comenius University Bratislava, Bratislava, Slovakia; 2 Institute of Zoology, Slovak Academy of Sciences, Bratislava, Slovakia; 3 Department of Biodiversity and Ecology, Plant Science and Biodiversity Centre, Slovak Academy of Sciences, Bratislava, Slovakia; 4 Institute of Fisheries of the National Academy of Agrarian Sciences of Ukraine, Kyiv, Ukraine

**Keywords:** alpha diversity, community structure, early flowering, eDNAmetabarcoding, pollinator competition

## Abstract

**Introduction:**

Plant-pollinator interactions may have positive, negative, or neutral influences on pollination and reproductive success of co-occurring plants. Some plants are highly attractive for pollinators (magnet species) and their presence can be beneficial for neighboring plants in terms of increased pollinator availability.

**Methods:**

Combining field and laboratory data, we examined pollinator visitation and reproductive success in early blooming co-occurring native plants, two-leaf squill (*Scilla* spp.) and snowdrop (*Galanthus nivalis* L.).

**Results:**

The blue flowers of *Scilla* significantly outcompeted the abundant white flowers of *G. nivalis* for pollinator visits. These differences were further supported by the higher abundance of pollinators found on *G. nivalis* petals (detected using eDNA metabarcoding) in experimental plots where *Scilla* was removed compared to plots where *Scilla* and *G. nivalis* co-occurred. eDNA analysis showed that the presence of *Scilla* significantly reduced alpha diversity and taxonomic richness of the *G. nivalis* pollinator community. Furthermore, the plot with *Scilla* showed a significant shift in community composition, with *G. nivalis* dominantly visited by taxa such as Lepidoptera, which may provide different pollination services, while the scilla-free group showed a more balanced and diverse composition including key Hymenoptera and Diptera taxa. This superior attractiveness of *Scilla*, consistent across both field observations and controlled lab experiments, strongly supports its role as a magnet species. Flower fertility of *G. nivalis* from plots with *Scilla* present was significantly lower than from plots without *Scilla*.

**Discussion:**

Overall, it appears that early flowering plant communities face strong competition for pollinators, which are scarce due to low spring temperatures. Magnet species may more significantly influence pollinator activity “in their favor” and potentially threaten the reproductive success of other, co-occurring species.

## Introduction

Flowering plants are the most diverse group of plants, actually with around 295,000 described species (angiosperms; monocots: 74,273; eudicots: 210,008, Christenhusz and Byng, 2016), and majority of them depends on pollination by animals (Ollerton et al., 2011). A mechanism of pollination basically involves movement of pollen from male parts to the female parts through an animal pollinator who benefits from by feeding on food rewards (most frequently nectar and/or pollen) provided by the flower. This mutualistic relationship is beneficial for both actors – the flower and for the pollinator (Proctor et al., 1996).

Many plants are pollinated by the same pollinators and have similar flowering periods and time of pollen and nectar presentation. If such species grow intermixed or near each other, they compete for the pollinators (Levin and Anderson, 1970). Plants may outcompete co-occurring species by an increased attractiveness of their flowers to pollinators by flower colour (Dafni et al., 1990; Ômura and Honda, 2005; Lázaro et al., 2009; Reverté et al., 2016), flower size (Conner and Rush, 1996; Knauer and Schiestl, 2017; Lobo et al., 2016; Spaethe et al., 2001), scents (Benelli et al., 2017; Dobson and Bergstrom, 2000; Flamini et al., 2007; Schäffler et al., 2015) or inflorescence shapes (Gómez et al., 2008a; b; 2009; Jersáková et al., 2012; Lázaro et al., 2009; Møller and Sorci, 1998). As a result, the evolution of these attractive traits is often driven by the selective pressure of interspecific competition for pollinator services.

Mechanisms of pollinator attraction play crucial role in interspecific competition, because pollinator’s visitation frequency influences the fertility of flowers (Bauer et al., 2017; Klein et al., 2003; Mallinger and Prasifka, 2017; Steffan-Dewenter and Tscharntke, 1999). In turn, pollinator availability in temperate zone is low particularly in early spring when temperature is low (Arroyo et al., 1985; Hirao et al., 2006; Totland and Matthews, 1998; Vicens and Bosch, 2000), and/or when flower abundance is high (Essenberg, 2012; Feinsinger et al., 1991; Nishikawa, 1998; Rathcke, 1983), thereby increasing the importance of flower preferences by pollinators.

Some highly attractive plants (magnet species, Thomson, 1982) have been shown to increase the pollinator service to other neighboring species (Molina-Montenegro et al., 2008; Muñoz and Cavieres, 2008; Feltham et al., 2015; Johnson et al., 2003; Laverty, 1992; Seifan et al., 2014). For instance, the deceptive orchid *Anacamptis morio* (L.) R.M. Bateman, Pridgeon & M.W. Chase experiences higher pollination success when growing among rewarding nectar plants (Johnson et al., 2003), and mayapple (*Podophyllum peltatum* L.) colonies show increased fruit and seed set when located near prolific nectar-producing lousewort (*Pedicularis canadensis* L.) (Laverty, 1992).

On the other hand, some evidence suggests that negative reproductive interactions between native plants can cause reduction in fertility for plants neighboring the magnet species (Thomson et al., 2019) and can result in character displacement in the breeding system (Fishman and Wyatt, 1999). This negative outcome occurs when more attractive species monopolize pollinators, increase detrimental heterospecific pollen deposition, or alter pollinator behavior unfavorably. Crucially, these competitive effects are often density-dependent. For example, while a few individuals of the invasive dandelions (*Taraxacum* spp.) may have a neutral effect, high densities of them have been shown to reduce native seed set by drawing visits away from neighbors. Similarly, experiments have shown that the facilitative effect of conspicuous neighbors can switch to competition as their density or aggregation increases (Muñoz and Cavieres, 2008; Seifan et al., 2014). This suggests that plant-pollinator interactions may have positive (facilitative), negative (competitive), or neutral influences on pollination and, consequently, on reproductive success of one or both interacting species (Rathcke, 1983).

eDNA is a modern method used to detect DNA traces left by flower visitors to identify pollinators and associated arthropods in terrestrial plants. It expands detectability and the temporal/spatial window of plant–pollinator interactions and can recover diverse arthropod assemblages—including small, cryptic, parasitic or nocturnal visitors—that are missed by visual/camera surveys (Johnson et al., 2023; Thomsen and Sigsgaard, 2019). This approach has proven particularly effective for detecting rare and elusive taxa such as small Diptera, Thysanoptera, and parasitoid Hymenoptera that are difficult to observe through traditional methods (Bell et al., 2017). eDNA metabarcoding can detect pollinator visits hours to days after the interaction occurred, as DNA persists on floral surfaces despite environmental exposure (Peel et al., 2019). Comparative studies have demonstrated that eDNA methods detect significantly higher arthropod richness and diversity than observational surveys, with some studies reporting 2–3 times more taxa through molecular approaches (Macgregor et al., 2019). The method has been successfully applied across diverse ecosystems, from agricultural landscapes to natural habitats, significantly helping to uncover pollination networks, crop pest dynamics, and biodiversity monitoring (Pornon et al., 2016).

In this study, we investigated pollinator-mediated interactions between two early-blooming plants: two-leaf squill (*Scilla* spp.) and snowdrop (*Galanthus nivalis*). These native species co-occur and often flower in high densities in Central European deciduous forests. We examined their competition for pollinators by combining field data on pollinator visitation rates with lab assessments of flower fertility. We also analyzed the spectral properties of the flowers to understand how pollinator vision influences flower preference. Given that bee pollinators, key early-spring visitors, typically exhibit a strong innate preference for blue colours over white in the UV-blue-green trichromatic visual system (Chittka et al., 1994; Dyer et al., 2016), we reasoned that floral colour could be a decisive factor in this system. We therefore hypothesized that the blue-flowered *Scilla* would act as a competitive magnet species, drawing pollinator visits away from the co-flowering, white-flowered *Galanthus nivalis*, despite its potentially lower local density. To test this, we established field plots where *Scilla* was either present or removed. We then assessed *G. nivalis* fertility and identified flower visitors by analyzing eDNA traces left on *G. nivalis* flowers using metabarcoding. We predicted that: (1) *G. nivalis* flowers in plots with *Scilla* present would receive fewer pollinator visits than those in *Scilla*-removal plots, and (2) consequently, *G. nivalis* seed set would be lower in plots where *Scilla* was present.

## Methods

### Study organisms

This study was carried out on flowers of the snowdrop (*G. nivalis*) with white, and the two-leaf squill (*Scilla* spp.) with blue oblong flowers belonging to the families *Amaryllidaceae* and *Asparagaceae*, respectively. Both species have herbaceous perennial growing from an underground bulb with radially symmetrical, insect pollinated flowers (Aschan and Pfanz, 2006; Zuraw, 2011). In conditions of Western Slovakia, flowering of both species occurs since late February to the end of March (P. Prokop, pers. obs.). There are both *Scilla vindobonensis* Speta and *S. bifolia* L. in Vlčkovský háj locality, while only *S. vindobonensis* is present in Bažantnica (see below). Because these two squill species are very similar in appearance, we refer to them as *Scilla* in the text.

### Study area

Observations on pollinator visits to *Scilla* and *G. nivalis* were recorded at two deciduous forest sites in spring 2015: Bažantnica (48.08190°N, 17.08956°E) and Vlčkovský háj (48.28639°N, 17.64955°E), both in Western Slovakia (~50 km apart). Four observation surveys were conducted, two at each site, between March 19 and 25. Only sunny, windless days with actual temperature around 14 – 17 °C were chosen for research in order to maximize insect visitation rates. All *G. nivalis* populations covered extensive areas and consisted of thousands of aggregated individuals. In contrast, *Scilla* is distributed individually among *G. nivalis* (Prokop et al., 2024). In the study areas as well as in surrounding habitats, *Scilla* always co-occurs with the *G. nivalis* and never forms coherent crops.

### Observations of bee visitation

To analyze the differences in pollinator visits, we tracked individual, randomly chosen honeybees until their first visit to any flower. Although we recorded the presence of other pollinators, non-bee visitors (mostly dipterans and hymenopterans) comprised a very low proportion (<5%) of all observed visits and were therefore excluded from the statistical analysis. Following Patiño et al. (2002), we considered a visit to be a legitimate pollination visit only when the honeybee remained on the flower for at least 3 s and made contact with the reproductive structures. This criterion was applied to exclude incidental visits for behaviors such as thermoregulation or rest. An individual bee was abandoned by the observer immediately after flower visitation to avoid pseudoreplication. Immediately after the honeybee started to feed on *G. nivalis*, we measured the nearest *Scilla* flower distance with tape meter to the nearest of 1 cm. If the honeybee visited *Scilla*, we measured the nearest *G. nivalis* flower distance. All measurements were performed between 11:30 and 15:30, when pollinators are usually the most active.

### Flower preferences in the laboratory

A single captive colony of naïve bumblebees (*Bombus terrestris*, L.), meaning they had no prior foraging experience with flowers, was procured from Koppert^©^ (Nové Zámky, Slovakia), and was kept at 22–24 °C in a room lit by natural light and neon light (370 lx). The bumblebees were connected to a 90 × 50 × 40 cm insectarium by a plastic mesh tube and daily fed with honey solution (60% water, 40% honey). Plastic gates in the tube allowed us to control the entry and exit of individuals into the flight cage. Foraging bees were tested individually by closing the entry of the captive colony. Honey solution was removed from insectarium and one freshly collected *G. nivalis* and *Scilla* flowers were placed in glass test-tubes 10 cm apart from each other on the rear part of the terrarium, which previously served as feeding place, 5 cm apart from the back wall. The placement of the flowers was randomly determined (i.e., left or right). We recorded preference for each of the two simultaneously presented flowers. Each bee and each flower was used only once. The trials (N = 20) took place 1 week after the colony arrived, in March 2022, during 09:00–14:30, indoors at the same conditions as described for keeping the colony. New flowers were used for each trial.

### Behavioral competition for pollinators between *G. nivalis* and *Scilla* in the field

Interspecific competition for pollinators was examined by comparisons of *G. nivalis* fertility between plots with and without presence of *Scilla*. Eight 10 × 10 m plots 10–30 m far from each other were established in February 2022. Four randomly selected plots were treated as experimental, and four plots served as control groups. All plots were visited two-times per week since February 22 till May 12, 2022. All flower buds of *Scilla* were systematically removed from the experimental plots, while control plots remained intact. A total of randomly selected 30 *G. nivalis* flowers were marked with a ribbon at the beginning of the experiment, and all produced capsules were collected and returned to the laboratory in May 2022.

### Competition for pollinators between the *G. nivalis* and *Scilla* in the field analysed with eDNA metabarcoding

In 2023, the experiment was repeated in the same localities. To minimize disturbance from tourist traffic, eight new 10 × 10 m plots were established approximately 10 m farther from a nearby path than those used in 2022. The experimental design, plot visitation schedule, and procedure for the systematic removal of *Scilla* flower buds in experimental plots (with controls remaining intact) were identical to those described in the 2022 experiment. All plots were visited two-times per week since February 22 till end of March 2023. In the mid of March 18–21 individual *G. nivalis* flowers were sampled from each of the eight locations. The S+ group, consisting of four locations (SG1, SG3, SG5, SG7) where *G. nivalis* grew in close proximity to *Scilla*, and the S- group, comprising four locations (SG2, SG4, SG6, SG8) where *Scilla* was removed. The eDNA metabarcoding, targeting the cytochrome c oxidase I (COI) gene, from DNA extraction to the identification of operational taxonomic units (OTUs), was performed following the protocol described in Prokop et al. (2024).

### Experimental impact of pollinator availability on plant fitness

To test whether absence of pollinators impacts fitness of *G. nivalis*, we marked undeveloped flower buds, covered them with fine mesh and 5–8 days after flowering begun the flowers, each representing different plant, were randomly divided to four groups:

1. The xenogamy treatment (n = 20 flowers); the stigma was pollinated with a brush, using pollen from an unfamiliar plant growing at least 10m apart from the focal plant.
2. Facilitated autogamy (n = 20 flowers); flowers were self-pollinated with a brush with pollen from the same flower.
3. Spontaneous autogamy (n = 20 flowers); flowers were left untreated. After pollination, all plants from these three groups were covered with fine mesh to avoid pollination by insects.
4. Control group (n = 20 flowers); flowers were left untreated and uncovered with fine mesh to facilitate pollination by insects.

### Measurement of the reflectance spectra

Spectral data provide an objective way to quantify colour (Endler 1990; Cuthill et al., 1999). We obtained reflectance spectra in the range 300–700 nm from randomly chosen tepals of *G. nivalis* (N = 20) and *Scilla* flowers (N = 20) using a spectrophotometer (OceanOptics WS-1-SS, a DT-Mini-2GS light source, and a QR400- 7-SR-BX bifurcated optic fibre) calibrated to a white standard. Reflectance was measured with the probe fixed in a RHP-1 holder at an angle of 45° to the tepal surface. We then calculated 1) the bee color space hexagon, where perceived color differences of stimuli are calculated based on the sensitivity of bee photoreceptors (Chittka, 1992; also validated for other hymenopterans, see Dyer and Chittka, 2004), and 2) the fly color space model, which relies on the sensitivity of blowfly photoreceptors and considers stimuli distinguishable between, but not within, four sectors of the dipteran color space (Troje, 1993; Arnold et al., 2009).

### Bioinformatic and statistical analysis

The metabarcoding data analysis was conducted in Taxon Table Tools (TTT) and R software v4.4.3 environment (R Core Team, 2025). For analysis and visualization, specialized R packages were used: vegan (for diversity analysis and PERMANOVA), ggplot2 (for data visualization), dplyr and tidyr (for data manipulation), indicspecies (for identifying indicator species), and VennDiagram (for visualizing Venn diagrams). To ensure the reliability of downstream analysis, OTUs identified as non-pollinator organisms (e.g., humans, microorganisms, fungi, nematodes, annelids or some non-pollinator arthropods like mites, water bears, psicids or predaceous and soil dwelling beetles) were initially excluded from the dataset. The presence of these species on flowers is not illogical; they could have landed on the flowers, been passively carried there, or their DNA could have gotten onto the flowers from the soil), but it is very unlikely that they participate in the pollination proces.

An analysis of the read distribution was performed following Noël et al. (2021). Read counts were sorted by OTU and sample, OTUs accounting for <0.05% (corresponding to max. 5 reads) of the total reads in a single sample were excluded to mitigate probable sequencing noise and potential artifacts. To account for varying sequencing depths among samples, the data were normalized using the DESeq2 package.

Diversity analysis was conducted to evaluate both alpha and beta diversity. The Shannon Index, Simpson Index, and Species Richness were calculated on the filtered, non-normalized integer count data. The Wilcoxon test was used to determine the statistical significance of differences between the S+ and S- groups. For beta diversity, the normalized data were used. A Principal Coordinates Analysis (PCoA) based on Bray-Curtis dissimilarity was employed to visualize inter-sample differences, and a Permutational Multivariate Analysis of Variance (PERMANOVA) was conducted to test for statistically significant differences between the groups. Overall taxonomic richness was calculated and visualized at various taxonomic levels. The distribution of shared and unique OTUs between groups was identified and visualized using a Venn diagram. Comparative “butterfly plots” were generated to visualise relative abundance of taxa. The most abundant OTUs were further analyzed via heatmaps to visualize their distribution across locations and groups. Group specific OTUs were identified using multipatt function of the indicspecies R package.

A general linear mixed model with Gamma error distribution was used the analyze the distance of the nearest flowering individuals of *G. nivalis* or *Scilla*. Plan species and site identity were used as explanatory variables, additionally site identity was controlled by the random factor. Two models were fitted, with the interaction term of explanatory variables and without the interaction term to analyse the potential site specific differences. Likelihood Ratio Test was used to compare the two models. R packages lme4, effects, lsmeans, ggplot2 and gridextra were used for data analyses and data visualisation.

## Results

### Pollinator visitation in the field

**Figure 1:**
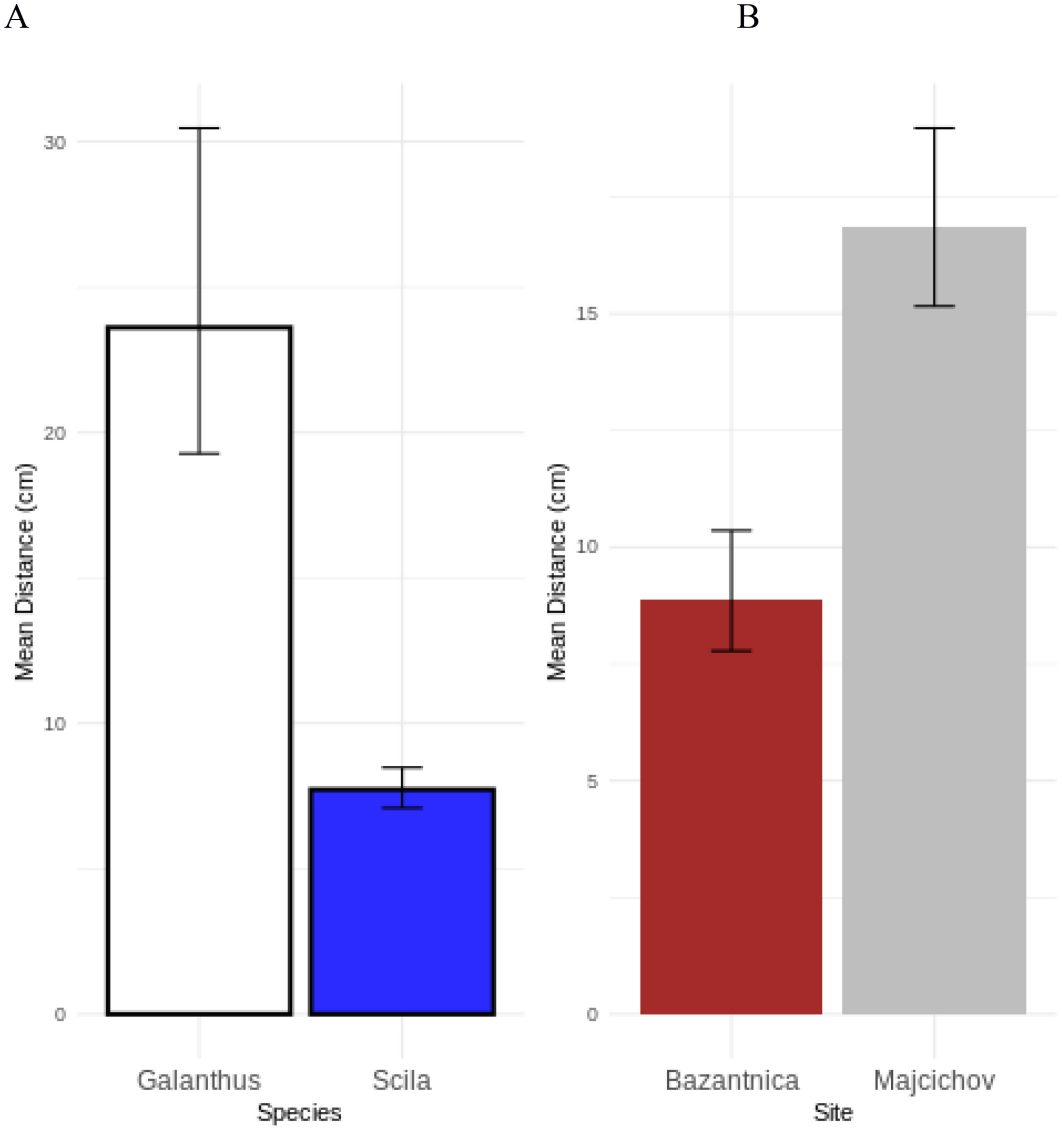
Mean nearest flower distance (cm) with respect to plant species **(A)** and study site **(B)**. Snowdrops (*Galanthus*) were visited by honeybees particularly when the distance of the two-leaf squill (*Scilla*) was long and vice versa **(A)**. Nearest flower distances were closer in Bažantnica (Bazantnica) compared with Vlčkovský háj (Majochov) **(B)**.

**Figure 2:**
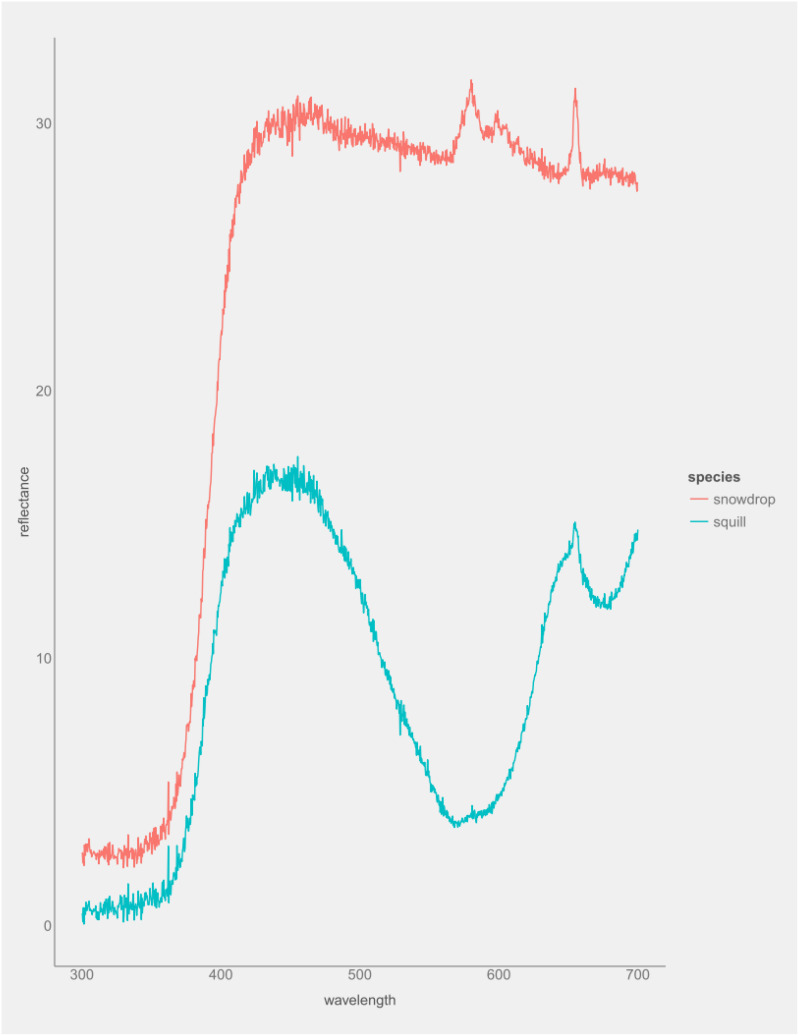
Spectral reflectatnce analysis of the two-leaf squill (*Scilla*) and the snowdrop (*G. nivalis*).

*Scilla* was visited by honeybees more frequently (230/263, 87.5%) than *G. nivalis* (33/263, 12.5%) (binomial test, *P* < 0.0001). Furthermore, flowers of *G. nivalis* were visited particularly when the distance of the nearest *Scilla* was longer (**Figure 1**). In contrast, close distance between *G. nivalis* and *Scilla* obviously resulted in pollination of the latter species (**Figure 1**). Based on the modelling procedure using GLMM with Gamma error distribution, the effect of species and site were important. The distance of the nearest *G. nivalis* was lower compared to the distance of the nearest *Scilla* (**Figure 2**). Also, the distances were different between sites, but the site specific pattern for both species was the same as interaction of both variables was not significant (Chisq=0.8185, df=1, p=0.3656).

### Flower preferences in the laboratory

There was clear preference for *Scilla* flowers by foraging bumblebees. Most individulas (18/20, 90%) preferred *Scilla* over *G. nivalis* flowers (binomial test, P < 0.001). While we acknowledge the modest sample size (N = 20), the effect size was exceptionally large (Cohen’s h = 0.93). The 95% confidence interval [69.9%, 97.2%] demonstrates that even the most conservative estimate shows a strong preference (>2:1 ratio). Bayesian analysis yielded a Bayes Factor of 262.8, indicating extreme evidence of preference over the null hypothesis of no preference.

### Competition for pollinators between *G. nivalis* and *Scilla* in the field

Control plants of *G. nivalis* with *Scilla* presence produced significantly lower number of capsules (11/120, 9.16%) than *G. nivalis* from experimental plots where *Scilla* flowers were removed (33/120, 27.5%) (Chi-square test, χ2 = 13.47, P = 0.0002). Total number of collected *G. nivalis* capsules per plot ranged between 1 and 11 (M = 5.5, SD = 3.85). The number of seeds per capsule was mean = 15.07 seeds (95% CI: [11.31, 18.83] N = 28 for treatment with *Scilla* removed and mean = 15.57 seeds (95% CI: [8.39, 22.75] for treatment with *Scilla* present. The Negative Binomial GLMM with ID of plot as random effect showed no treatment effect (coefficient = 0.033, p = 0.908) on number of seeds per capsule. The 95% CI for the treatment effect [-0.521, 0.587] includes zero, confirming no difference between treatments. Gamma distribution GLMM with treatment as predictor and capsule ID and plot ID as random effects showed no differences in mean weight of seeds between plots with *Scilla* (M = 0.0091 g, SD = 0.0047 g, N = 108) and plots without *Scilla* (M = 0.0097 g, SD = 0.0136 g, N = 422) (estimate = −0.0845 on log scale; multiplicative effect ≈ 0.919, 95% CI ≈ [0.57, 1.49], P = 0.732).

### Experimental impact of pollinator availability on plant fitness

We found no capsules in treatments with spontaneous (0/20) and induced autogamy (0/20), but xenogamous plants produced 7/20 (35%) capsules. Each capsule contained seeds. Control plants produced comparable number of capsules (6/20, 30%) containing seeds. Differences in abortion rates were significant (Fisher exact test, P < 0.001), suggesting that pollen/pollinator availability significantly improves reproductive success on *G. nivalis*. There were no significant differences in mean capsule weight between xenogamous (median = 0.33, 95% CI 0.19 – 0.61, N = 7) and control plants (median = 0.48, 95% CI 0.23 – 0.69, N = 6) (M-W U-test, U = 17.0, P = 0.62).

### Spectral reflectance analysis of flower tepals

*G. nivalis* consistently shows much higher reflectance (peaking around 30–32%) across the visible spectrum compared to *Scilla* (peaking around 15%) (**Figure 2**). *Scilla* flowers have lower reflectance throughout, with a maximum around 15% and a pronounced dip in the green-yellow region. With respect to UV region, *G. nivalis* reflectance rises sharply from ~3% at 300 nm to over 30% by 400 nm. *Scilla* also rises, but from near 0% to about 13% at 400 nm. This suggests that *G. nivalis* reflects UV more strongly than *Scilla*. Regarding blue spectrum, *G. nivalis* showed high, relatively flat reflectance (~30%), while *Scilla* peaks in blue (~15% at 450 nm), then declines. This suggest that *Scilla*’s blue peak may contribute to its blue coloration; *G. nivalis* high, flat reflectance suggests a white appearance. Regarding green-yellow spectrum, *G. nivalis* remains high (~30%), with minor fluctuations and *Scilla* shows a pronounced trough, dropping to ~5% reflectance around 570 nm. This suggests that *Scilla* may appear less reflective (darker) in green-yellow, enhancing blue coloration. With respect to red spectrum (600–700 nm), *G. nivalis* maintains high reflectance (~30%), with small peaks and the reflectance of *Scilla* rises again to ~13% at 700 nm. These results suggest that *G. nivalis* high reflectance across all visible wavelengths is consistent with a white flower; *Scilla* lower, variable reflectance is consistent with blue/purple hues.

### Bee color space analysis

**Figure 3:**
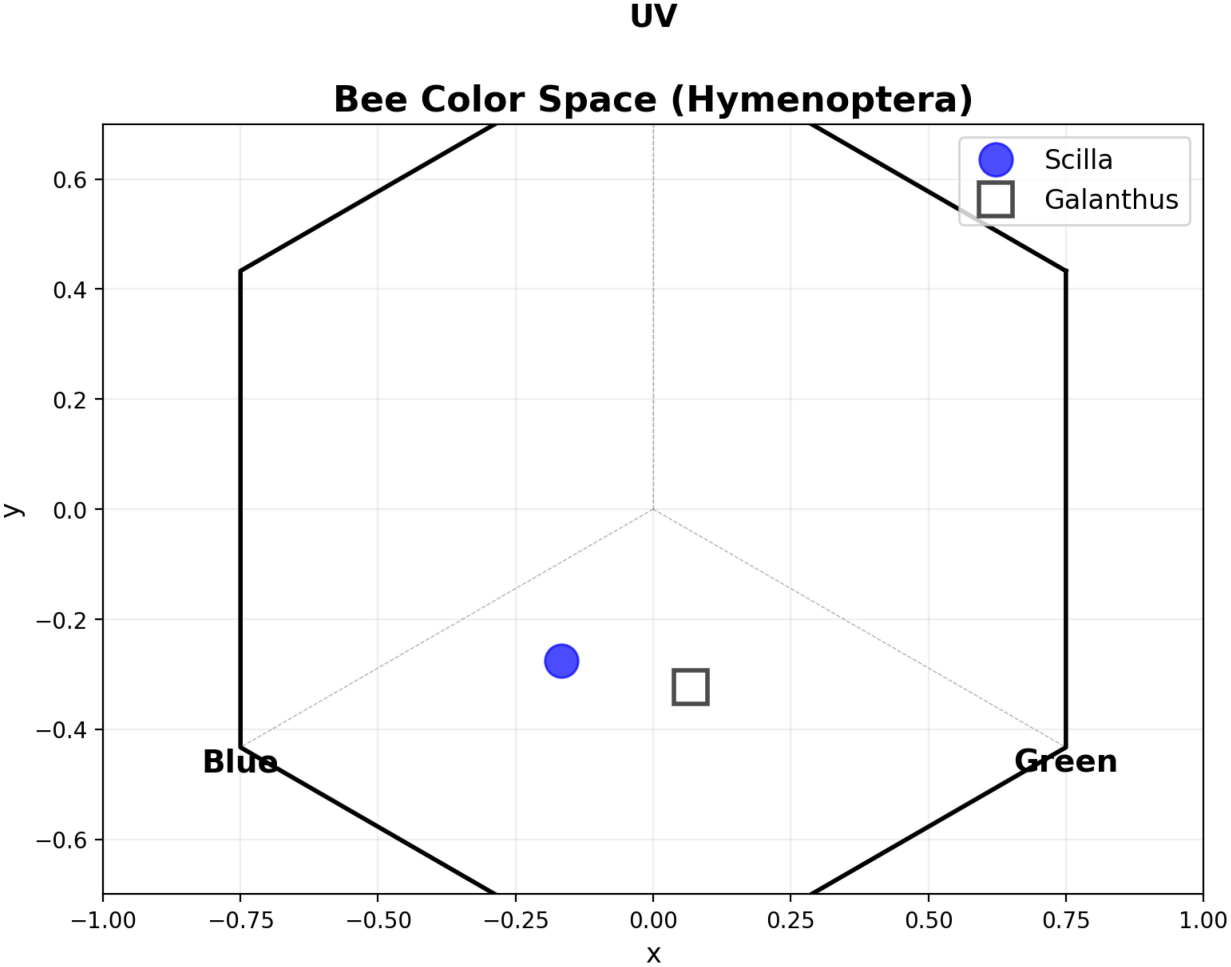
Hymenopteran color space showing the perceptual positions of *Scilla* (blue circles) and *Galanthus* (gray squares) flowers. The plot is divided into four color sectors based on opponent channels: Green - UV (x-axis) and Blue - Red (y-axis). *Scilla* flowers occupy the UV-Blue sector (Sector 1), while *Galanthus* flowers are positioned near the achromatic center.

The bee color hexagon shows how *Scilla* (blue circle) and *Galanthus* (white square) appear to hymenopteran pollinators like bees (**Figure 3**). Both flowers are positioned in the lower portion of the hexagon, indicating low UV reflectance, supporting previous descriptive analysis. The positions show both flowers stimulate blue and green photoreceptors more than UV. *Scilla* is positioned more toward the blue region (left side, x=-0.167), while *Galanthus* is shifted toward green (right side, x=0.068). This suggests that bees can distinguish between these two flower species based on their color signals, with *Scilla* appearing more blue-shifted and *Galanthus* appearing more green/white-shifted in bee vision.

### Fly color space analysis

**Figure 4:**
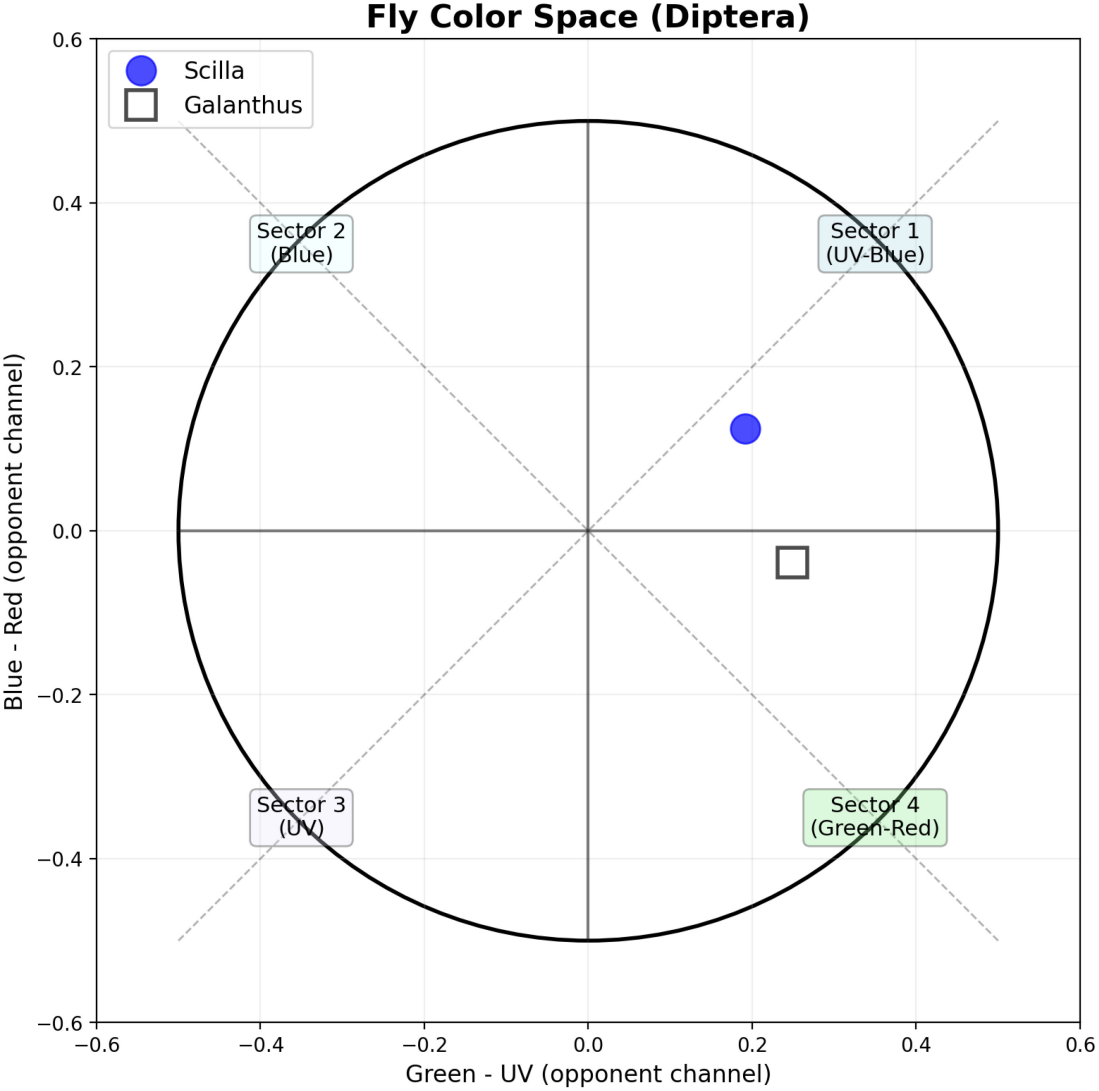
Dipteran color space showing the perceptual positions of *Scilla* (blue circles) and *Galanthus* (gray squares) flowers. The plot is divided into four color sectors based on opponent channels: Green - UV (x-axis) and Blue - Red (y-axis). *Scilla* flowers occupy the UV-Blue sector (Sector 1), while *Galanthus* flowers are positioned near the achromatic center.

The fly color space model divides the perceptual space into 4 categorical sectors. Both flowers fall in Sector 1 (UV-Blue region): *Scilla*: x=0.191, y=0.124 (upper right quadrant), *Galanthus*: x=0.249, y=-0.039 (right side, near horizontal axis) (**Figure 4**). According to Troje’s categorical model, stimuli are distinguishable between sectors but not within sectors. Since both plants fall within the same sector (Sector 1), flies may not reliably distinguish between these two flower species based on color alone. However, *Galanthus* is positioned very close to the boundary with Sector 4, suggesting it may appear slightly more green-red shifted to flies. These results suggest that these flowers may be more effectively targeted toward hymenopteran pollinators (bees) rather than dipteran pollinators (flies).

### eDNA metabarcoding analyses

**Figure 5:**
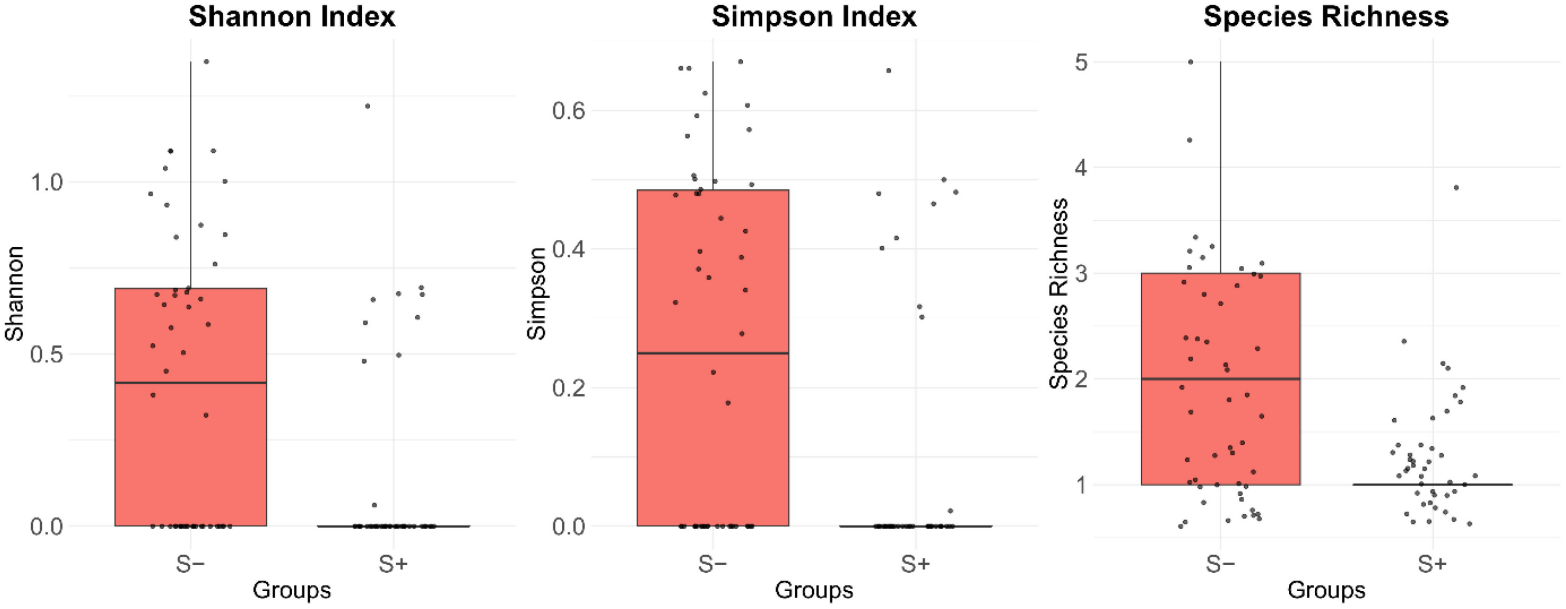
Comparison of alpha diversity metrics between the S- and S+ groups.

Multi-step filtration revealed 52 OTUs in 94 samples, which were used in downstream analysis. Alpha diversity analysis revealed significant differences in pollinator community richness and evenness between S+ and S- group (**Figure 5**). The S- group showed higher median values for the Shannon Index (P = 0.0012), Simpson Index (P = 0.0016), and Species Richness (P = 0.0007).

Analysis by individual location confirmed these patterns (**Supplementary Figure 1**). The S- group locations consistently exhibited higher median diversity values than locations of the S+ group.

**Figure 6:**
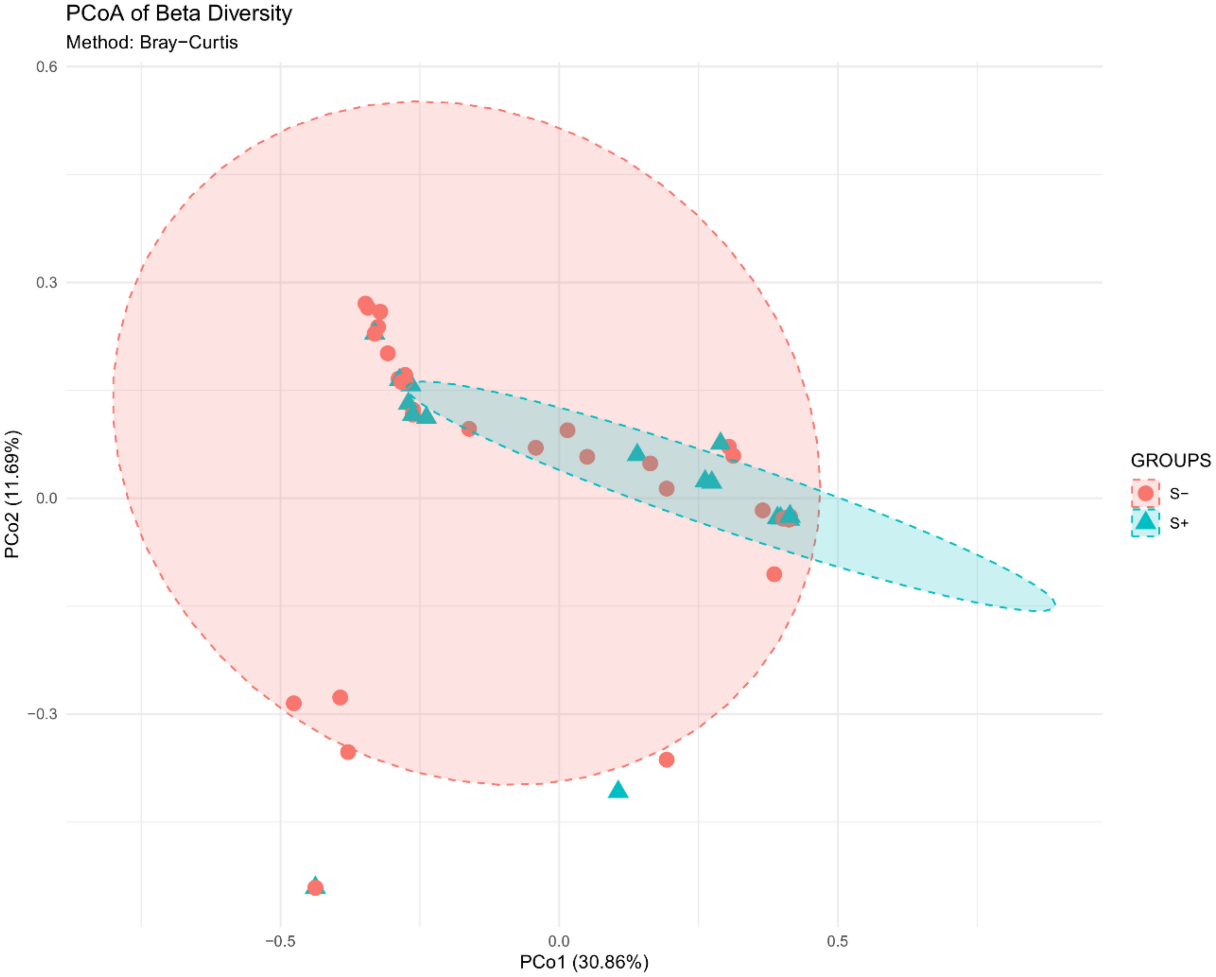
Principal Coordinates Analysis (PCoA) of beta diversity based on Bray-Curtis dissimilarity, showing distinct clustering between the S- and S+ groups.

PCoA analysis revealed distinct clustering between groups with PCo1 explaining 30.86% of the variation (**Figure 6**). PERMANOVA confirmed significant differences in community composition (F = 7.38, p=0.001, R²=0.075).

**Figure 7:**
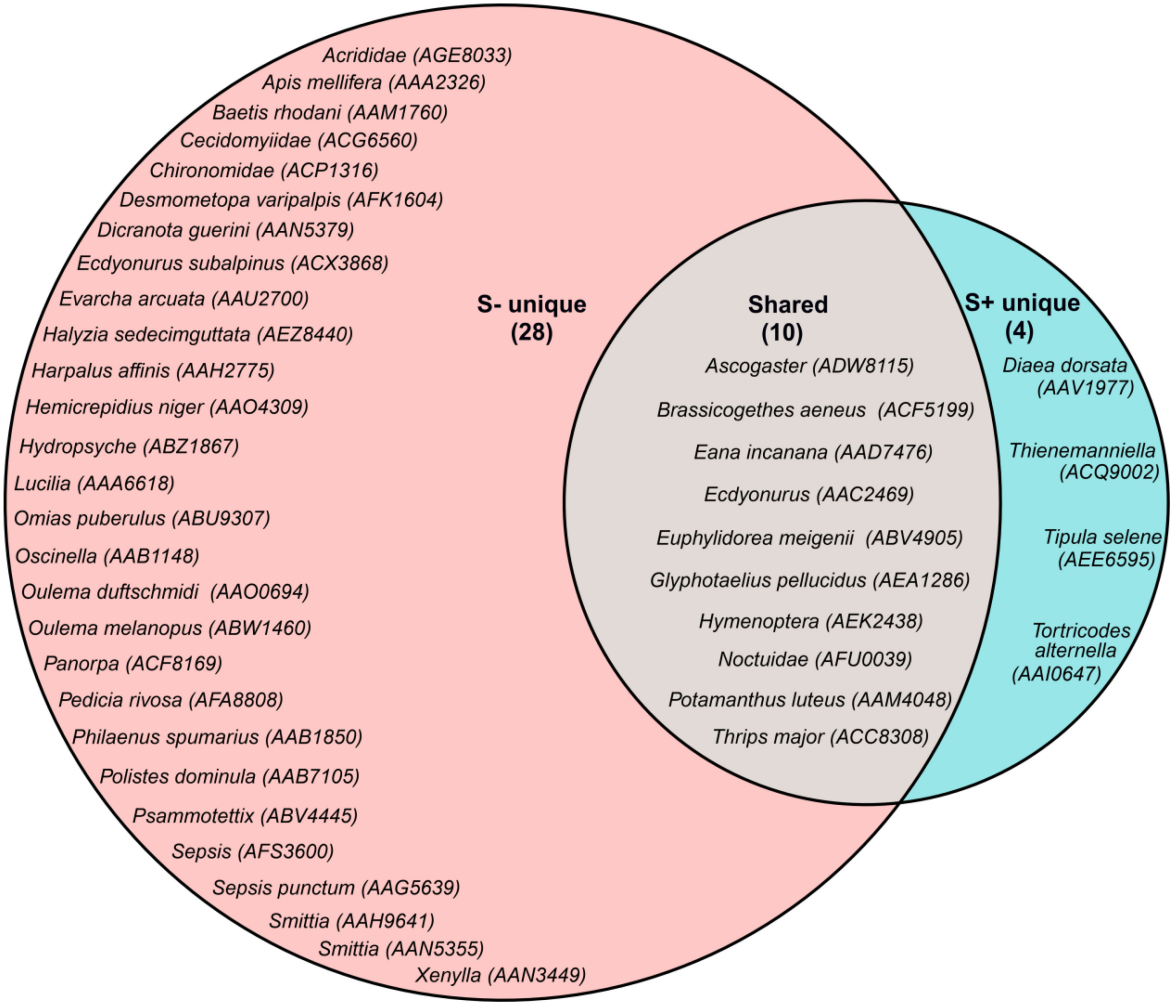
A Venn diagram illustrating unique and shared OTUs between the S- (Scilla-free) and S+ (Scilla-present) groups. The size of each circle is proportional to the number of OTUs. Specific taxonomic names and BOLD IDs are provided for each category.

The S- group demonstrated higher taxonomic richness at all levels. This pattern was consistent across individual locations (**Supplementary Figure 2**). At the OTU level, S- group locations ranged from 12 to 17 unique taxa per location, S+ group locations had 3 to 8 unique taxa per location. 28 OTUs were unique to S-, f unique to S+, and 10 shared OTUs (**Figure 7**).

**Figure 8:**
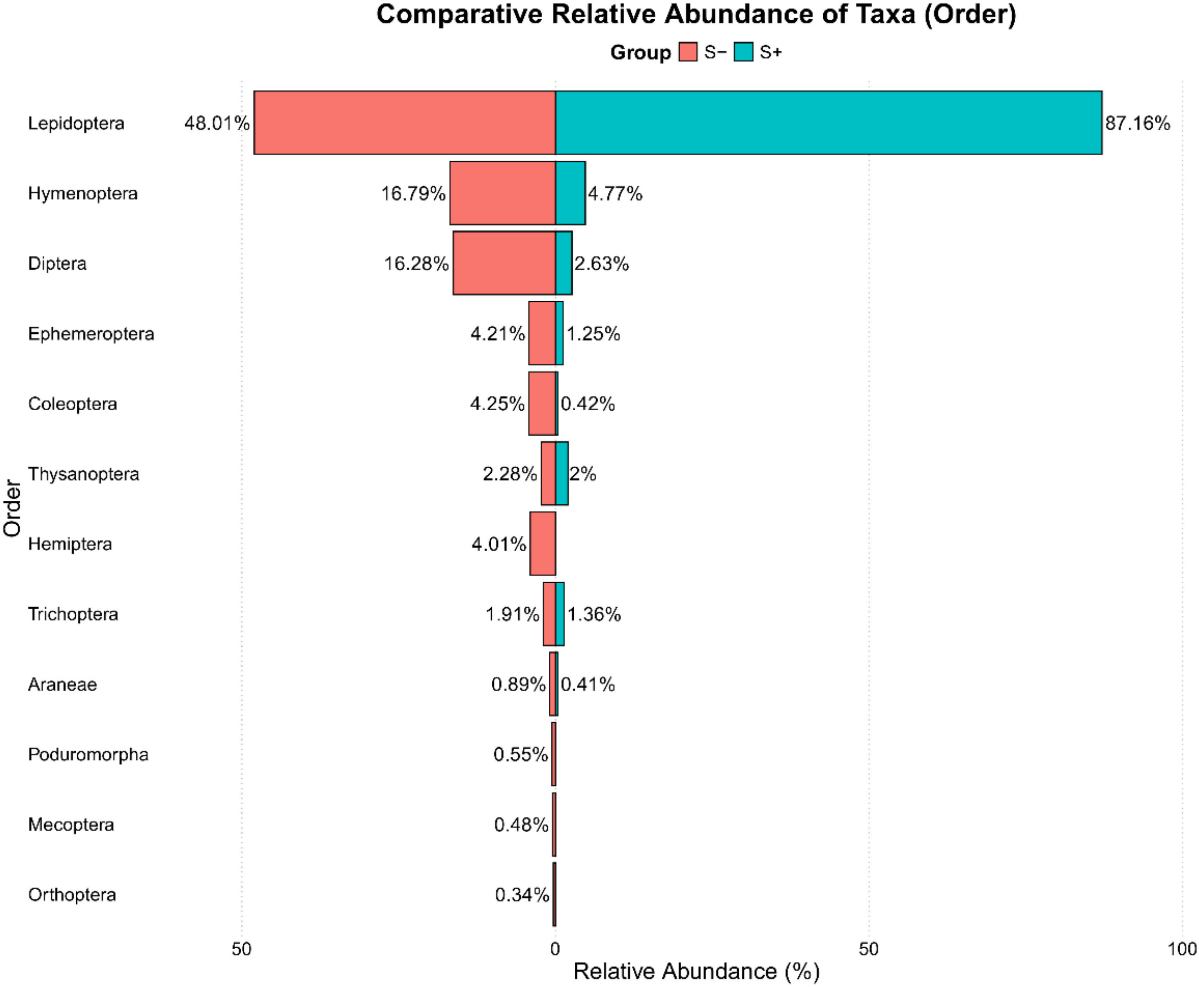
Comparative relative abundance of taxa at the order level between the S- and S+ groups, highlighting differences in community composition.

At the order level, the S+ group was dominated by Lepidoptera (87.16%). The S- group showed a composition where Lepidoptera accounted for 48.01%, Hymenoptera for 16.79%, and Diptera for 16.28% (**Figure 8**).

At the OTU level, *Eana incanana* (Stephens, 1852) (BOLD: AAD7476) dominated the S+ group (74.72%) compared to S- group (38.01%) (**Supplementary Figure 3**). Taxa such as *Apis mellifera* Linnaeus, 1758 (BOLD: AAA2326), *Philaenus* sp*umarius* (Linnaeus, 1758) (BOLD: AAB1850), and *Polistes dominula* (Christ, 1791) (BOLD: AAB7105) were found exclusively in the S- group. Indicator species analysis identified two significant indicators for the S- group: Noctuidae sp. (BOLD: AFU0039) (p=0.008) and Hymenoptera sp. (BOLD: AEK2438) (p=0.020).

**Figure 9:**
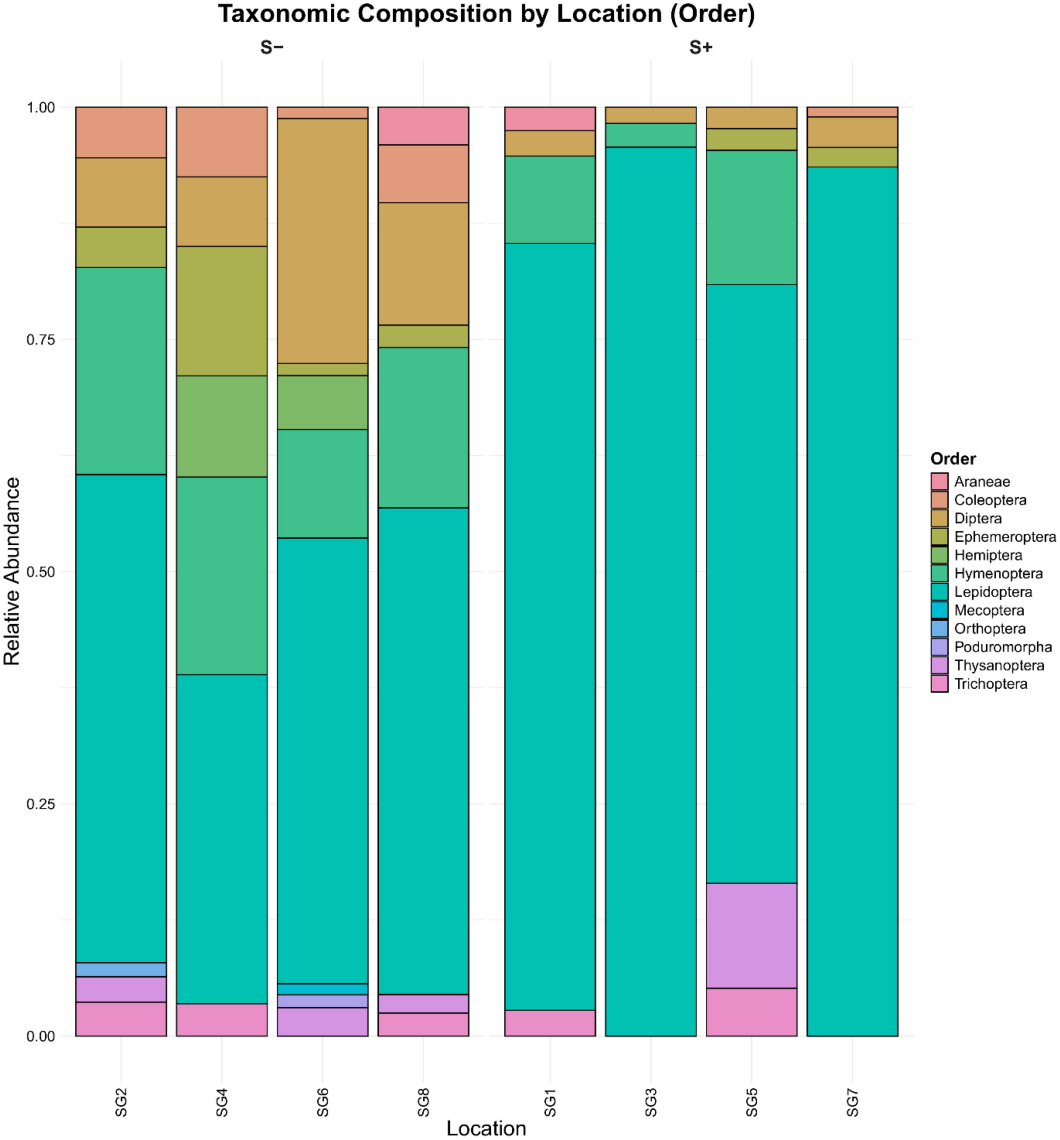
Taxonomic composition of pollinator communities by individual locations, showing relative abundance at the Order level for both S- and S+ groups.

Consistent with the significant differences in community composition confirmed by PERMANOVA, the analysis of taxonomic composition at each location showed consistent patterns (**Figure 9**). S+ group locations were dominated by Lepidoptera across all sites. S- group locations exhibited a more diverse taxonomic composition with more significant representation of Hymenoptera and Diptera.

## Discussion

This study investigated pollinator-mediated interactions between two co-occurring plants with similar phenologies. Both early flowering and high flower densities are associated with low pollinator availability (Arroyo et al., 1985; Hirao et al., 2006; Totland and Matthews, 1998; Vicens and Bosch, 2000), and thus, competition for pollinators could be expected. In line with this expectation, we found that flower visitation of honeybees, a model pollinator species available in early spring, was much higher in *Scilla* than in *G. nivalis*. These findings were supported by tracking honeybees in the field, by choice expriments with bumblebees in the laboratory and by analysing DNA leftovers of arthropods visiting *G. nivalis* using eDNA metabarcoding. These observations suggest that ecological interactions between these two species seem to be negative (competitive), rather than facilitative or neutral.

### Flower visitation

*Scilla* strongly outcompete *G. nivalis* flowers in attracting honeybees, because a wast majority of observations (87.5%) resulted in visiting flowers of the former species. Furthermore, *G. nivalis* flowers were visited by honeybees particularly when their distance from *Scilla* was longer. This suggests that *Scilla* can be considered as magnet species (Thomson, 1982) having negative impact on the neighboring *G. nivalis*. Campbell (1985) similarly found that experimental removal of *Claytonia virginic* flowers, which were frequently visited by pollinators, yielded to an increase of seed production in the co-occurring *Stellaria pubera* plant. Importantly, high densities of *G. nivalis* decrease the chance of individual flowers to be pollinated due to dilution effects (Goulson, 2000; Nielsen et al., 2012; Veddeler et al., 2006).

There may be several non mutually exclusive mechanisms of successful competition for honeybees favouring frequent flower visitation of *Scilla*. First, honeybees have visual system with three photoreceptors peaking in the UV (344 nm), blue (438 nm) and green (560 nm) regions of the spectrum (Chittka, 1992; Peitsch et al., 1992). Blue flowers of *Scilla* sp. fully correspond with an innate preference of honeybees for blue flowers (Arnold et al., 2009; Giurfa et al., 1995). However, blue coloration alone does not guarantee stronger bee preferences, as flower shape and reward accessibility are also important predictors of pollinator choices (Rentería and Brehm, 2025). We lack data on nectar amount and quality in snowdrops and *Scilla*, but the latter’s upward-facing flowers attract naïve bumblebees more than downward-facing ones (Prokop et al., 2020). *Scilla* may thus benefit from their attractive coloration and upright orientation, especially after favorable preceding weather, when nectar is undiluted and pollen is undegraded by rain (Lawson and Rands, 2019; Prokop et al., 2025). In contrast, snowdrops may offer a better option for pollinators on rainy or snowy days, as the rewards in their downward-facing flowers are less affected by rain than those in upward-facing flowers (Nakata et al., 2022; Prokop et al., 2023). Second, white background colour made by *G. nivalis* flowers can make flowers of *Scilla* even more visible compared with brown background produced by the ground lacking vegetation. Indeed, some colour contrasts show strong attractiveness to bees (Heiling et al., 2003) and honeybees are capable of learning objects on white background (Srinivasan, 1994).

eDNA metabarcoding revealed that Lepidoptera and Hymenoptera dominated as probable pollinators of *G. nivalis*, while Diptera were primarily visitors. These results are strongly supported either by our own observations (Diptera used flowers particularly for landing rather than as a food source) as well as by hexagon analyses. Honey-bee colour vision is based on excitation of UV, blue and green photoreceptors (Chittka, 1992). Lepidoptera, particularly within the Noctuidae family, possess trichromatic color vision, which is facilitated by the same three types of photoreceptors sensitive to different wavelengths of light, allowing these moths to detect a range of colors in their environment (Briscoe and Chittka, 2001; Kelber et al., 2002). Thus, Hymenoptera and Lepidoptera species are similarly able to discriminate between *G. nivalis* and *Scilla* sp. while the vision of Diptera (including red, ~620nm) is fundamentally different from moths and bees (Arnold et al., 2009; Troje, 1993). Thus, increased prefrences for *G. nivalis* in plots with absent *Scilla* sp. fits with our expectations. Lower abundance and diversity of insects on *G. nivalis* of S+ plots suggests that insects were not attracted by magnetic *Scilla* flowers and relied more frequently on less attractive *G. nivalis*. Finally, eDNA metabarcoding helped us to identify species with noctuids having nocturnal activity, which could not be observed by us, given that our field work was restricted to daytime. The competitive exclusion was quantitatively supported by beta-diversity analysis using eDNA data. Specifically, the PERMANOVA revealed that approximately 7.5% of the total variation in pollinator community composition was statistically explained by group membership (presence/absence of *Scilla* spp.). Furthermore, analysis of the pollinators at the order level revealed a significant shift in community structure. While the group without *Scilla* showed a more balanced and diverse composition, including a greater proportion of Hymenoptera (bees and wasps) and Diptera (flies), the pollinator community associated with *G. nivalis* in *Scilla*+ plots was dominated by Lepidoptera (moths). Given that many Hymenoptera, such as *A. mellifera*, which was detected exclusively in the S- group, are often highly effective pollinators, this shift suggests a change in the quality of the pollinator service. Our interpretation of pollinator efficiency is based on the established life history of the detected taxa; however, the specific implications for *G. nivalis* reproductive success would depend on the relative efficiency of the remaining Lepidoptera visitors, a factor that was not directly measured in this study. This molecular evidence of competitive exclusion offers a comprehensive and unbiased view of the entire pollinator community, confirming that the presence of the magnet species causes structural changes (a quality effect), and not just a reduction in visitation frequency.

### Flower fertility

*G. nivalis* is pollinator dependent (this study), although under some conditions it can be partially self-compatible (Weryszko-Chmielewska and Chwil, 2016). We showed that the number of seeds or their weight per capsule did not differ among plots, which is not surprising, given that these samples likelly came from pollinated flowers.

Most importantly, the number of capsules produced by *G. nivalis* was significantly lower in S+ plots, suggesting that the presence of *Scilla* possess reproductive costs for *G. nivalis*. The overall capsule production per plant was very low in all plots. Although this may not reflect a common pattern, it is likely attributable to the extremely dry spring during the study period. Water deficit is a critical factor that limits plant performance, as it intensifies competition for resources between vegetative and reproductive growth. Under such conditions, plant reproductive output may be reduced. This is consistent with the findings in other species, where drought stress leads to increased abortion of reproductive structures (e.g., *Eucalyptus globulus*; Suitor et al., 2010). Therefore, the observed low capsule numbers likely resulted from the combined effect of interspecific competition with *Scilla* and the acute environmental stress of the unusually dry season.

Even if we accept the fact that some *G. nivalis* are self-compatible (Weryszko-Chmielewska and Chwil, 2016), self-pollination is costly to plants, because such plants produce fewer seeds and/or inbred offspring suffer from reduced viability (Charlesworth and Charlesworth, 1987; Darwin, 1876; Herlihy and Eckert, 2002). Future research should examine whether the rate of self-pollination in *G. nivalis* decreases as the distance from *Scilla* increases.

### Limitation

A methodological consideration is that the repeated manual removal of *Scilla* flowers in S- plots could have influenced local plot conditions. Specifically, this activity has the potential to disrupt pollinator behavior through an increase in human presence. However, several lines of evidence suggest that this intervention did not impair plant-pollinator interactions in a way that biased our principal findings. First, human activity was not confined to S- plots; all plots (both S+ and S-) were visited with equal frequency during routine fieldwork, standardizing any generalized disturbance effect. Crucially, our eDNA metabarcoding data showed that *G. nivalis* flowers in S- plots (where *Scilla* was removed) carried a greater number of pollinator cues than those in S+ plots. This indicates that the plots with the most intensive human intervention (*Scilla* removal) experienced higher visitation rates to snowdrops, which is the opposite of the expected pattern if human presence deterred pollinators. The removal work was conducted daily between 09:30 and 11:00 hours. Pollinator activity in this early spring ecosystem was minimal during this window, with peak activity consistently occurring after 11:00. Therefore, while a minor disturbance effect cannot be entirely ruled out, it is unlikely to account for the observed competitive suppression of *G. nivalis* reproduction by *Scilla*.

Additionally, the laboratory choice tests relied on a single bumblebee colonies. This limits the generalizability of these behavioral results, as they do not capture the full behavioral and genetic diversity of the pollinators present in the field. Furthermore, our field eDNA data indicated that honeybees (*A. mellifera*) are a key pollinator, whereas the laboratory assays used bumblebees (*Bombus terrestris*) as a model system. This choice was made for practical reasons, as bumblebee colonies provide naïve foragers essential for controlled experiments and share a similar trichromatic visual system with that of honeybees. However, we acknowledge that future research directly involving honeybees would help fully validate the preference mechanisms inferred from our field data. Future research would benefit from using more genetically diverse model systems to validate these preference patterns in the wild.

## Conclusion

The snowdrop (*G. nivalis*) and the two-leaf squill (*Scilla*) seem to be in negative interaction, as both species depend on pollinators and bloom early in spring when pollinators are scarce. The results of this study received complementary support using behavioral observation in natural environment, laboratory choice tests and eDNA analyses of insect visitation on *G. nivalis* flowers. We hypothesize that *Scilla* may benefit from higher visibility of its blue flowers growing among abundant white *G. nivalis* flowers which may have reproductive costs on neighboring *G. nivalis*. To further elucidate the costs of pollinator competition, future work should investigate the resulting reproductive trade-off in *G. nivalis*. This would involve comparing the allocation to sexual reproduction versus clonal propagation under conditions with and without co-flowering competitors, thereby revealing how competition for pollinators shapes their reproductive strategy.

## Data Availability

The datasets presented in this study can be found in online repositories. The names of the repository/repositories and accession number(s) can be found in the article/**Supplementary Material**.

## Supplementary material

Supplementary Figure 1 Comparison of alpha diversity metrics for pollinator communities across different locations (S-: SG2, SG4, SG6, SG8; S+: SG1, SG3, SG5, SG7).

Supplementary Figure 2 Comparative taxonomic richness between locations across different taxonomic levels.

Supplementary Figure 3 Comparative relative abundance of taxa at the OTU level between the S- and S+ groups.
