# Supplementary material for: The magnet species effect of two-leaf squill (Scilla spp.) on pollinator competition with the snowdrop (Galanthus nivalis L.)
Source: Front Plant Sci. 2026 Jan 30;17:1760796. doi: 10.3389/fpls.2026.1760796 (PMC12903926; doi:10.3389/fpls.2026.1760796)
Supplement: Supplementary Figure 1 — Comparison of alpha diversity metrics for pollinator communities across different locations (S-: SG2, SG4, SG6, SG8; S+: SG1, SG3, SG5, SG7). [file DataSheet1.docx]

**Supplementary**


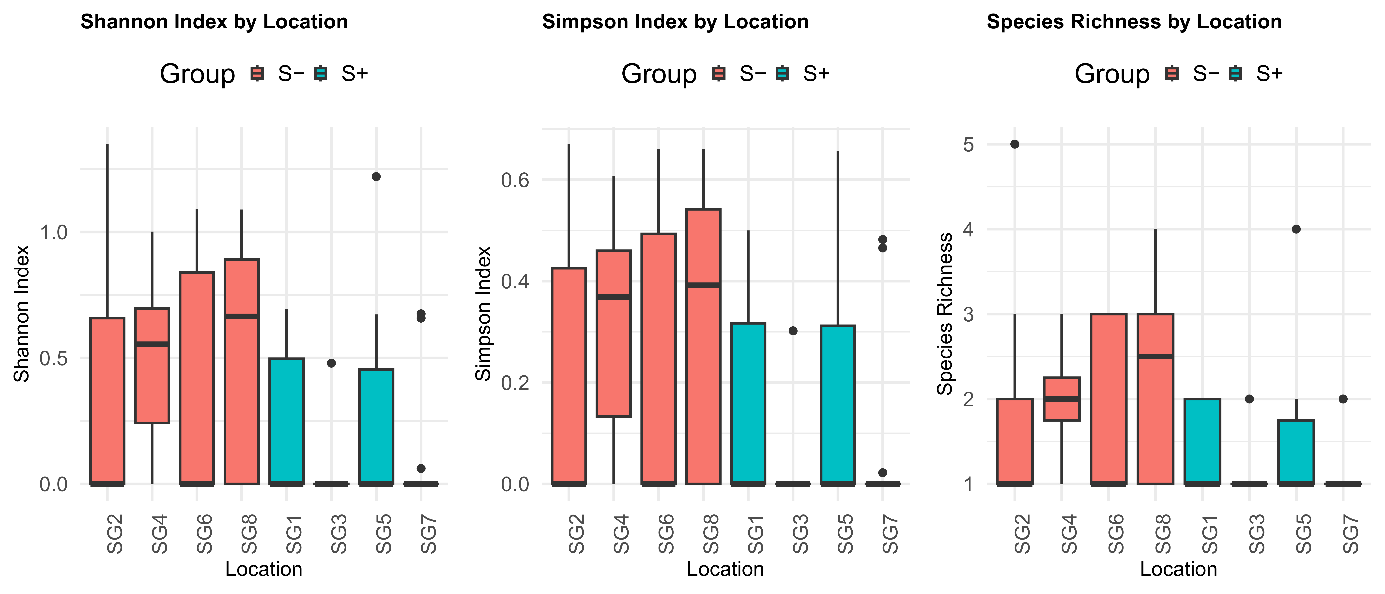


Suppl. Fig. 1. Comparison of alpha diversity metrics for pollinator communities across different locations (S-: SG2, SG4, SG6, SG8; S+: SG1, SG3, SG5, SG7).


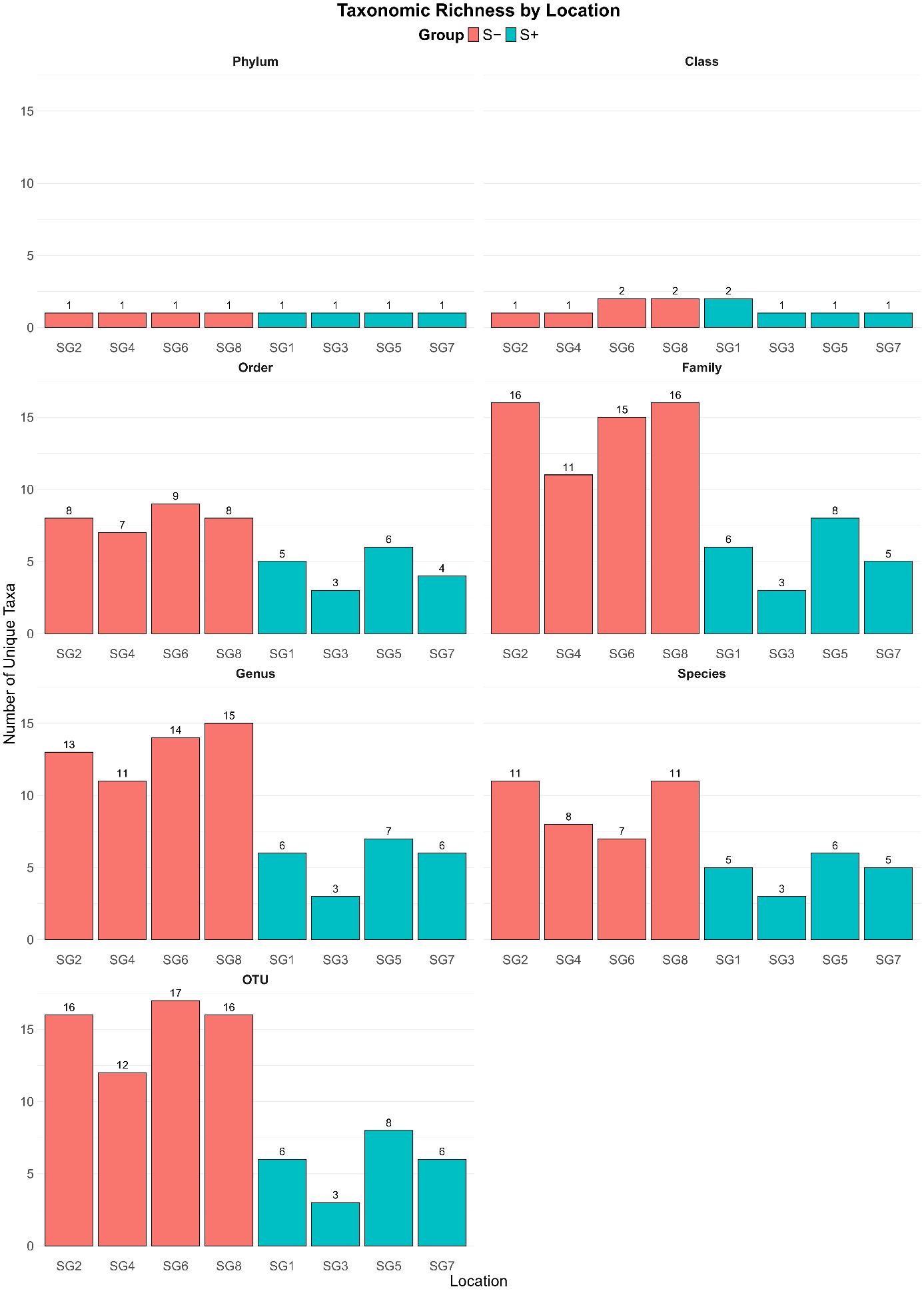


Suppl. Fig. 2 Comparative taxonomic richness between locations across different taxonomic levels.





Suppl. Fig. 3. Comparative relative abundance of taxa at the OTU level between the S- and S+ groups.
